# Compensatory Response of the Somatotropic Axis from IGFBP-2b Gene Editing in Rainbow Trout (*Oncorhynchus mykiss*)

**DOI:** 10.3390/genes11121488

**Published:** 2020-12-10

**Authors:** Beth M. Cleveland, Shiori Habara, Jin Oikawa, Lisa M. Radler, Munetaka Shimizu

**Affiliations:** 1 National Center for Cool and Cold Water Aquaculture, United States Department of Agriculture/Agricultural Research Service, Leetown, WV 25430, USA; lisa.radler@usda.gov; 2 Graduate School of Environmental Science, Hokkaido University, Sapporo 060-0808, Japan; diamonddust1181@eis.hokudai.ac.jp (S.H.); oijin@eis.hokudai.ac.jp (J.O.); 3 Faculty of Fisheries Sciences, Hokkaido University, Hakodate 041-8611, Japan; mune@fish.hokudai.ac.jp

**Keywords:** gene editing, CRISPR/Cas9, IGF, IGFBP, rainbow trout, feed deprivation, insulin-like growth factor

## Abstract

Rainbow trout with gene editing-induced reductions in serum insulin-like growth factor binding protein (IGFBP)-2b exhibit similar growth performance compared to fish without IGFBP-2b gene disruption. The objective of this study is to determine how the components of the insulin-like growth factor (IGF)/IGFBP system respond to a reduction in serum IGFBP-2b abundance. Editing the IGFBP-2b genes in rainbow trout resulted in an 83% decrease in serum IGFBP-2b in mutants. This resulted in a 35% reduction in serum IGF-I, which was offset by reduced expression of hepatic *igfbp-1a2* and increased muscle *igfr-1a*; these responses suggest that an increased IGF-I signaling capacity offset reductions in serum IGF-I. During feed deprivation, the differential expression of *igfbp* genes supports the attenuation of the growth inhibitory response, likely due to the further reduction in serum IGF-I that alleviated the need for an IGF-inhibitory response. Unique *igfbp* expression patterns occurred during refeeding, suggesting an enhanced IGF-I signaling capacity in controls. Collectively, these findings support that the role of IGFBP-2b is to regulate serum IGF-I concentrations. The compensatory regulation of IGF/IGFBP system genes indicates that adjustments in other IGFBP, both circulating and at the local level, maintain IGF-I signaling at a level appropriate for the nutritional state of the fish.

## 1. Introduction

Insulin-like growth factor (IGF) is recognized as a central regulator of anabolic growth in vertebrates [1], largely due to its positive effects on growth-related mechanisms in muscle [2,3,4] and bone [5,6]. Growth hormone (GH)-stimulated production of hepatic IGF-I is largely responsible for regulating the circulating levels of IGF-I, although local production of IGF-I and IGF-II is also acknowledged as a significant contributor to IGF signaling [7]. The majority of IGF-I is not free in circulation; in humans and salmonids, approximately 99% of IGF-I is bound to IGF binding proteins (IGFBP) [8,9]. In addition to extending the half-life of IGF-I in serum, IGFBPs function to sequester IGF-I to peripheral tissues and can promote or inhibit ligand binding to surface receptors [10,11]. In vertebrates, there are six IGFBP members (IGFBP-1 through IGFBP-6) and, due to a lineage-specific whole genome duplication, teleosts have two paralogs for each IGFBP member [12,13]. A second whole genome duplication event in salmonids ultimately resulted in 19–22 IGFBP genes [14]. 

In salmonids, the IGFBP subtypes IGFBP-1a, IGFBP-1b, and IGFBP-2b are detected in serum [9,15,16], each encoded by duplicated genes (IGFBP-1a1/IGFBP-1a2, IGFBP-1b1/IGFBP-1b2, and IGFBP-2b1/IGFBP-2b2). Of the three subtypes, IGFBP-2b is found in the greatest abundance and binds an estimated 80% of the IGF-I in serum [9]. The regulation of these IGFBP during physiological challenges such as feed deprivation and refeeding [17,18] or exposure to sex steroids [19] are often more dramatic and precede the IGF-I response, suggesting that the regulation of IGFBP abundance is a significant mechanism regulating IGF-I signaling in fish. In salmonids, IGFBP-1 subtypes are considered as growth inhibitory, since increases in gene expression and serum abundance are reported during catabolic conditions [20,21,22], while IGFBP-2b may promote IGF-I signaling by forming a stabilizing complex with IGF-I [11]. In salmonids, the expression of *igfbp-2b* and *igf1* is often directionally coordinated [23,24], suggesting that maintaining an appropriate equilibrium between these proteins is significant for regulating a physiological response. 

Characterizing the functions of the IGFBP subtypes is essential to understand the role of these proteins as regulators of IGF signaling. The application of gene editing technology, such as the Clustered Regularly Interspaced Short Palindromic Repeats (CRISPR)/Cas9 system, is useful for this initiative, as loss-of-function studies can be valuable for characterizing biological processes [25]. Previously, we described the application of CRISPR/Cas9 technology in rainbow trout; fish with bi-allelic mutations in the IGFBP-2b1 and IGFBP-2b2 genes were produced with reductions in circulating IGFBP-2b by up to 95% compared to wild-type controls [26]. Body weight and fork length of the mutants was slightly reduced early in development but did not differ from the controls at one year of age, indicating that the reduction in plasma IGFBP-2b did not cause sustained effects on growth. However, it was expected that the mutants would exhibit a growth benefit due to elevated free IGF-I and subsequent increased IGF-I signaling. It was hypothesized that compensatory responses occurred within the somatotropic axis that maintained IGF-I signaling at a comparable level between mutants and controls; this was evidenced in part by a reduction in circulating IGF-I in mutants. However, it is unknown how other axis components, such as locally produced IGFBP, IGF receptors, or IGFs, contribute to the compensatory response.

The objective of this study is to determine how components of the somatotropic axis are affected by the disruption of a functional IGFBP-2b protein by gene editing in rainbow trout. We subject controls and mutants to three feeding regimes (continuous feeding, feed deprivation, refeeding) that induce the regulation of the somatotropic axis to determine if the mutant phenotype is affected by this physiological perturbation. The findings reveal compensatory regulation of somatotropic components in mutants that support the maintenance of IGF-I signaling at a level appropriate for nutritional status.

## 2. Materials and Methods

### 2.1. Microinjection and Early Rearing

All procedures involving live fish were reviewed and approved by the National Center for Cool and Cold Water Aquaculture Institutional Animal Use and Care Committee (Protocols #115 and #153). Eggs and milt from two-year-old rainbow trout broodstock were collected and stored in ziplock bags at 4 °C and used for microinjections within one day of collection. Eggs were fertilized, rinsed, then stored in milt activator (102.8 mM NaCl, 10 mM Tris, 20 mM glycine, pH 9.0, 1 mM reduced glutathione) in a 10 °C incubator and microinjected within 2–7 hours post fertilization. Microinjections were performed using a previously described protocol [26]. Eggs were microinjected with one of two ribonucleotide-protein (RNP) complexes composed of gene-specific crRNAs, tracrRNA, and Cas9 protein (IDT Technologies, Coralville, IA). One RNP complex contained three crRNAs to produce IGFBP-2b mutants; two crRNAs independently targeted IGFBP-2b1 and IGFBP-2b2, and one crRNA targeted the TYR2 gene. The second RNP complex contained TYR2 crRNA and was used to produce the microinjected control group. The sequences of the crRNAs are as follows: TYR2: TGCTGCCCGGTGTGGGAGG (sense); IGFBP-2b1: ACACACCGAGGTGTTCCACA (sense); IGFBP-2b2: CTGCCGGTTCTATTGCTCGG (sense). The disruption of the TYR2 gene induced partial to complete albinism due to the loss of melanin production, therefore albinism served as a phenotypic tracer for gene disruption in both the control and mutant treatment groups [27]. Approximately 1350 eggs were injected for each the control and mutant treatment groups. The same number of un-injected eggs were retained to determine the effects of microinjection on the hatch rate. The average hatch rate of un-injected eggs was 42%, while the hatch rate of the injected groups averaged 34%.

The egg groups were hatched individually and transferred to six L buckets for early rearing. Just after first feeding, the mutants were combined with an equal number of controls to avoid tank effects on growth. At approximately 5 months post hatch, the fish (15–20 g, *N* = 806) were anesthetized with methane tricaine methanesulfonate (MS-222, 100 mg/L), tagged with passive integrated transponders (PIT tag), and the adipose fin was removed and stored in ethanol for genomic DNA extraction. Fish remained comingled until the start of the feeding study. Fish were hand fed during the fry stage (Finfish Starter, Zeigler Bros Inc, Gardners, PA, USA) and transitioned to automatic feeders that dispensed feed (Finfish G, Zeigler Bros., Inc) at a fixed percent of tank biomass that was just below satiation. 

### 2.2. Identifying IGFBP-2b Mutants via PCR and Ligand Blotting

**Figure 1:**
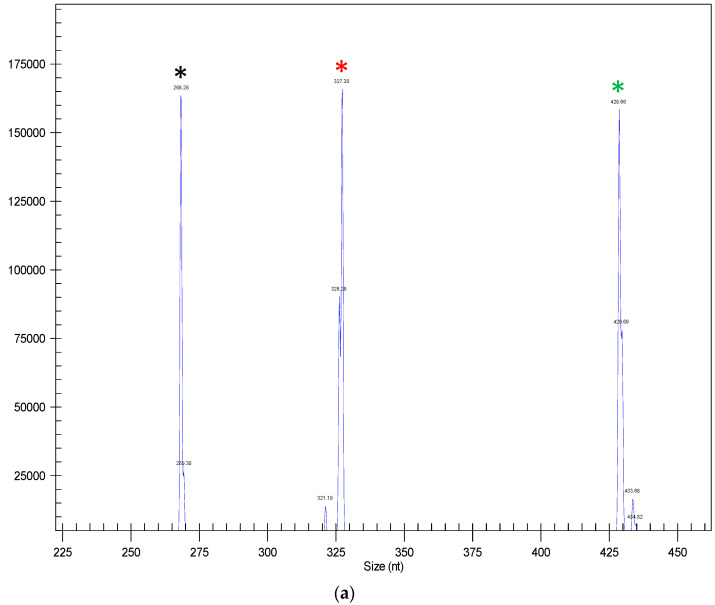

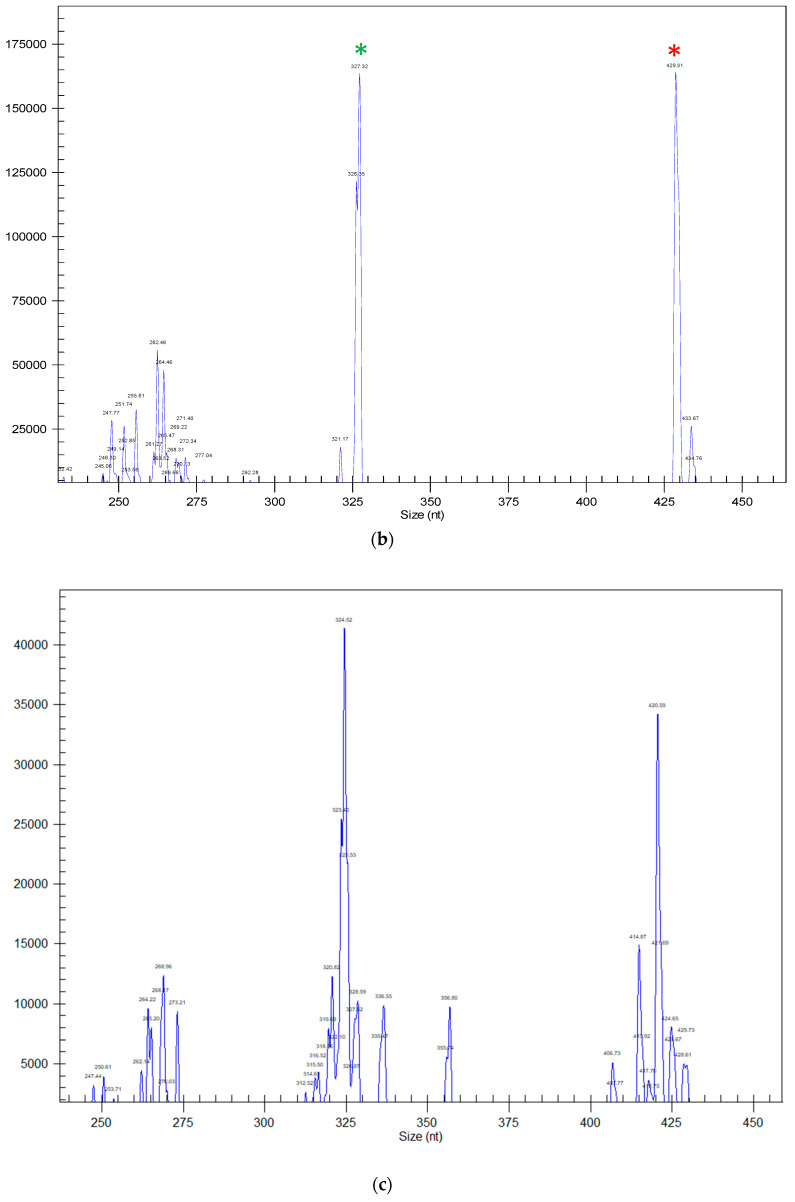

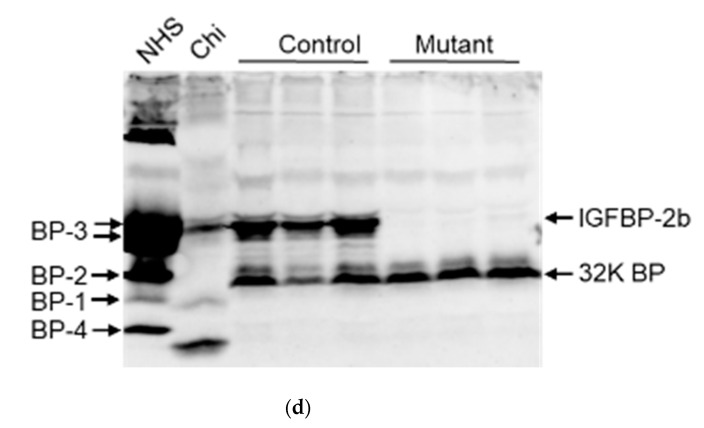
Panels a through c represent examples of chromatograms, indicating amplicons from (**a**) un-injected fish (**a**), injected controls (**b**), and high-rate IGFBP-2b mutants (**c**). Black asterisk indicates intact TYR2, red asterisk indicates intact IGFBP-2b1, and green asterisk indicates intact IGFBP-2b2. Panel (**d**) shows a representative Western ligand blot indicating IGFBP abundance in serum from control and IGFBP-2b mutant rainbow trout. Arrows on the left indicate known IGFBP in normal human serum (NHS). The arrows on the right indicate IGFBP-2b and a 32 kDa IGFBP in rainbow trout. Chi: Chinook salmon.

Genomic DNA was isolated using an AutoGenprep 965 according to the manufacturer’s recommended protocol. Gene mutagenesis was detected as previously described [26] by PCR followed by the separation of amplicons using capillary gene electrophoresis on a GeXP Genetic Analysis System (Beckman Coulter, Brea, CA, USA). Forward primers (Table S1) contained fluorescent tags for amplicon detection. Intact genes were identified as single peaks at ~268 bp (TYR2), ~327 bp (IGFBP-2b1), and ~428 bp (IGFBP-2b2). Gene mutagenesis was identified as multiple peaks (indels) in place of a single amplicon at the expected length. Injected controls were characterized as having a disrupted TYR2 but intact IGFBP-2b1 and IGFBP-2b2, while the mutants exhibited indels for each of the three genes. The PCR analysis qualified 209 control fish (26% of total) with severe TYR2 disruption and intact IGFBP-2b (Figure 1b) and 192 mutant fish (24% of total) with severe disruption of IGFBP-2b1, IGFBP-2b2, and TYR2 genes (Figure 1c). 

Western ligand blotting with digoxigenin-labeled human IGF-I (DIG-hIGF-I) was performed according to a previously described protocol [28]. Serum IGFBP levels were normalized to the human IGFBP-4 band intensity and expressed as arbitrary density units (ADU). Example ligand blots indicating IGFBP-2b protein abundance in controls and mutants are provided in Figure 1d. Mutants that were defined by the near-complete or complete disruption of IGFBP-2b1, IGFBP-2b2, and TYR2 and displayed the albino phenotype were selected for the study. Similarly, controls were selected that displayed the albino phenotype. Mutants displayed an average 83% reduction in serum IGFBP-2b abundance compared to controls.

### 2.3. Experimental Design and Sample Collection

#### 2.3.1. Feeding Study

Eighty-four mutant and 84 control fish (approximate age: 7 months) were weighed and equally divided between 6 tanks (150 L) per fish group (*N* = 12 tanks, *n* = 14 fish per tank). Fish were acclimated to experimental tanks for one week and hand-fed 1% of tank biomass twice daily, followed by hand feeding to satiation approximately one hour after the last feeding. At the beginning of the study, all the fish were anesthetized (MS-222, 100 mg/L) and the body weights and lengths were recorded. Three tanks per group continued satiation feeding for the entire four-week trial. The remaining six tanks were feed-deprived for three weeks then fed to satiation during the fourth week. At the end of week three and four, all the fish were analyzed for weight and length. In addition, six fish per tank were euthanized with a lethal dose of anesthetic (MS-222, 300 mg/L) and blood was collected from caudal vasculature and stored on ice until processing. Livers were excised and weighed. Liver and white muscle samples were frozen in liquid nitrogen prior to storage at -80 °C. After the sampling was complete, the serum was separated by centrifugation (2000 cpm, 10 min, 4 °C) and stored at −80 °C.

#### 2.3.2. 15 Months Post-Hatch Analysis

A cohort of the control (*n* = 15) and mutant (*n* = 15) fish used for the experiment described above were comingled in a single tank and reared in a similar manner as the continually fed treatment group described above. At approximately 15 months post-hatch, the fish were anesthetized and the body weights and fork lengths were recorded. Approximately 3 mL of blood was removed from caudal vasculature and stored on ice. Serum was separated by centrifugation (2000 cpm, 10 min, 4 °C) and stored at −80 °C prior to the analysis of serum IGF-I (total and free) and IGFBPs.

### 2.4. Sample Analysis

#### 2.4.1. Plasma IGF-I Analysis

Plasma IGF-I was quantified according to a previously published time-resolved fluoroimmunoassay [29], with minor modifications, using recombinant trout IGF-I and anti-barramundi IGF-I antiserum (GroPep, South Adelaide, Australia). 

#### 2.4.2. Free IGF-I

Serum-free IGF-I was separated from bound IGF-I using ultrafiltration by centrifugation, as previously described [30]. Serum (200 µL) was applied to an anisotropic, hydrophilic ultrafiltration membrane mounted in the Centrifree device (EMD Millipore, Billerica, MA, USA) and centrifuged at 1000× *g* at 15 °C for 30 min. One hundred microliters of the ultrafiltrate was lyophilized, reconstituted in 4 µL water, and subject to the acid-ethanol extraction to ensure recovery.

#### 2.4.3. Gene Expression

Gene expression analysis was performed using conventional protocols that have been previously described [31]. Briefly, RNA was isolated and cDNA was produced using random primers and M-MLV reverse transcriptase. The PCR utilized SYBR Green with an ABI7900 Sequence Detection System (Applied Biosystems, Foster City, CA, USA). A melt curve was performed for each reaction to confirm a single peak. Gene expression was normalized to the geometric mean of two reference genes (beta actin and elongation factor-1a) using Genorm software. Primers were designed to separately amplify transcripts from duplicated genes, with the exception of gene duplicates with a high sequence similarity (igf1, igfbp-2a, igfbp-4, igfbp-5a, igfr-1a). Primer sequences and amplification efficiencies are provided in Table S1. 

### 2.5. Statistical Analysis

Statistical analysis was performed using PC-SAS (Version 9.2) (SAS, Cary, NC, USA). An analysis of variance (ANOVA) was used to detect the main effects. In the event of a significant effect, a Tukey’s test was used for multiple means comparisons. To detect differences between the controls and mutants during continuous feeding, a two-way ANOVA was performed to test for the main effects and interactions between (1) the treatment group and (2) the experimental week. A one-way ANOVA was used to detect gene regulation due to feeding treatment (feed deprivation or refeeding); each ANOVA was performed separately for controls and mutants. In addition, differences in the magnitude of regulation between controls and mutants that were either feed deprived or refed were detected using one-way ANOVA. All fold changes in gene expression were log_2_-transformed prior to statistical analysis. Regression and Pearson correlation analyses were performed using SigmaPlot software. Free IGF-I levels were log transformed prior to Pearson correlation analysis.

## 3. Results

### 3.1. Growth Response

**Figure 2:**
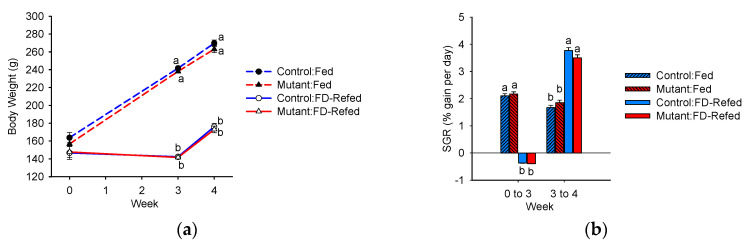
Body weight (**a**) and specific growth rate (SGR) (**b**) over the course of the trial. Differences between treatment groups within the same experimental week are indicated by different letters (*p* < 0.05, *n* = 21 or 42). FD: feed deprivation.

Average body weight did not differ between controls and mutants at the beginning of the study or during feed deprivation and refeeding (Figure 2a). As expected, feed deprivation-induced weight loss and recovery growth was observed during refeeding that was evidenced by greater SGR compared to continuously fed fish (Figure 2a,b). The numerical difference in SGR between the controls and mutants after refeeding was not statistically significant (*p* = 0.09). Feed intake, fork length, and condition factor were similar between the control and mutant fish within the same feeding treatment (data not shown), supporting the concept that there is not a morphological phenotype associated with IGFBP-2b disruption. 

### 3.2. Responses in Continuously Fed Fish

**Figure 3:**
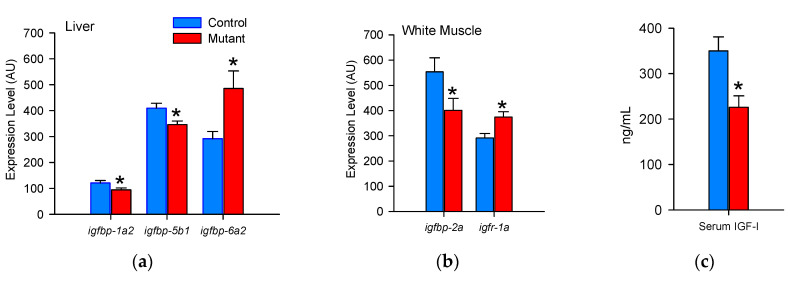
Differential regulation of somatotropic axis components. Panels indicate differentially regulated genes in (**a**) liver and (**b**) white muscle and (**c**) serum IGF-I in continuously fed control and mutant fish. Asterisks indicate significantly different means, *p* < 0.05 (gene expression: *n* = 21; plasma IGF-I: *n* = 8). AU: arbitrary units.

Genes that were differentially expressed between continuously fed controls and IGFBP-2b mutants are shown in Figure 3. In liver, mutant fish exhibited a reduced expression of *igfbp-1a2* and *igfbp-5b1* and a greater expression of *igfbp-6a2* compared to the controls (Figure 3a). In white muscle, only *igfbp-2a* and *igfr-1a* were differentially regulated between the control and mutant fish, in which mutants displayed lower and higher expression, respectively (Figure 3b). Mutants also exhibited an approximately 35% lower serum IGF-I concentration compared to the controls (Figure 3c).

### 3.3. Feed Deprivation and Refeeding Response

#### 3.3.1. Serum IGF-I and IGFBP

**Figure 4:**
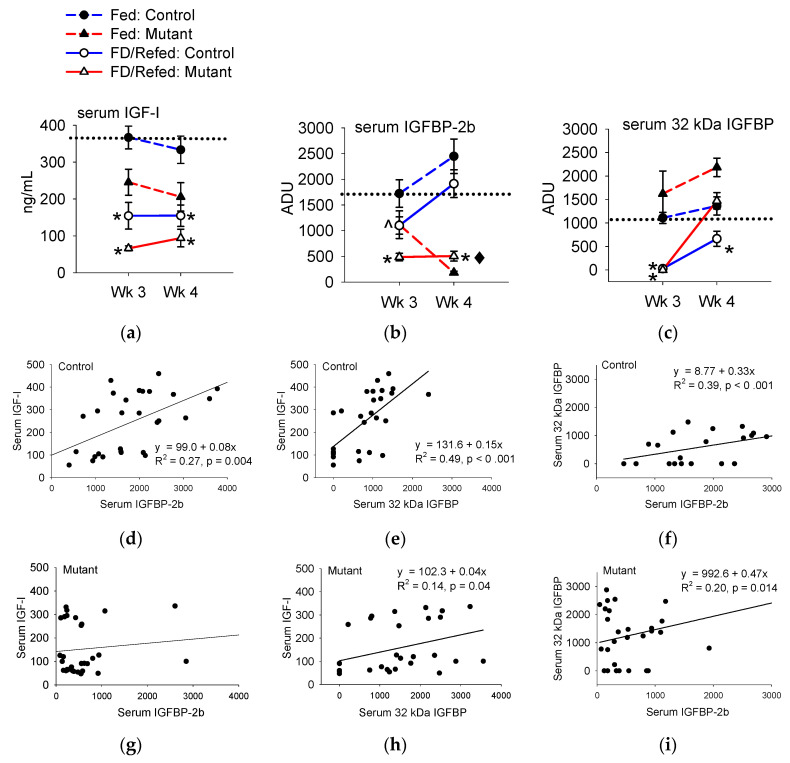
Abundance of serum (**a**) IGF-I, (**b**) IGFBP-2b, and (**c**) 32 kDa IGFBP during feed deprivation (FD, Wk 3) and refeeding (Wk 4). The horizontal dotted line is a visual reference for the mean of the continuously fed control fish at Wk 3. Asterisks indicate that the feed-deprived (dashed line) or refed groups (solid line) differ from continually fed fish within the same treatment group and week. The carrot (^) in panel (**b**) represents *p* = 0.06. The black diamond indicates that the magnitude of regulation from continually fed fish differs between the mutants and controls. Panels (**d**) through (**i**) represent associations between plasma variables in control (**d**–**f**) and mutant (**g**–**i**) fish. Trendlines indicate the regression of best fit; analysis data are provided when *p* < 0.05 (*n* = 8).

During feed deprivation, the concentrations of serum IGF-I in controls and IGFBP-2b mutants were reduced to approximately 0.3-fold of continuously fed fish (Figure 4a, Table S2). The concentrations of IGF-I in feed-deprived mutants (66 ng/mL) were lower (*p* < 0.05) than in controls (155 ng/mL). Serum IGF-I remained reduced after one week of feed deprivation and was lower (*p* < 0.05) in mutants (94 ng/mL) compared to the controls (155 ng/mL). The abundance of serum IGFBP-2b is reported in place of transcript abundance, since transcript stability can be affected by gene mutagenesis due to nonsense-mediated mRNA decay mechanisms. Relative to continuously fed fish, the abundance of IGFBP-2b in serum was only numerically reduced in the controls by 38% (*p* = 0.065) during feed deprivation and by 23% during refeeding (*p* = 0.15). The abundance of IGFBP-2b in mutants is significantly lower (35–92%) than in controls within the same feeding treatment (*p* < 0.05) (Figure 4b).

An IGFBP band at 32 kDa was detected in the plasma of controls and mutants, and its intensity was reduced by feed deprivation. Refeeding restored the 32 kDa IGFBP in controls but not in mutants (Figure 4c).

#### 3.3.2. Correlations among IGF-I and IGFBPs

Control and mutant serum samples were pooled within feeding groups to characterize the relationship between serum response variables. There was a positive linear relationship between serum IGFBP-2b abundance and IGF-I concentrations in the control fish (Figure 4d), while there was no association between these variables in the mutants (Figure 4g). The IGF-I concentrations were also positively correlated with serum 32-kDa IGFBP abundance in both controls (Figure 4e) and mutants (Figure 4h), although the coefficient of determination was low in mutants. There was a positive relationship between serum IGFBP-2b abundance and 32-kDa IGFBP abundance in both groups (Figure 4f,i).

#### 3.3.3. Expression of *igf* and *igfr*

**Figure 5:**
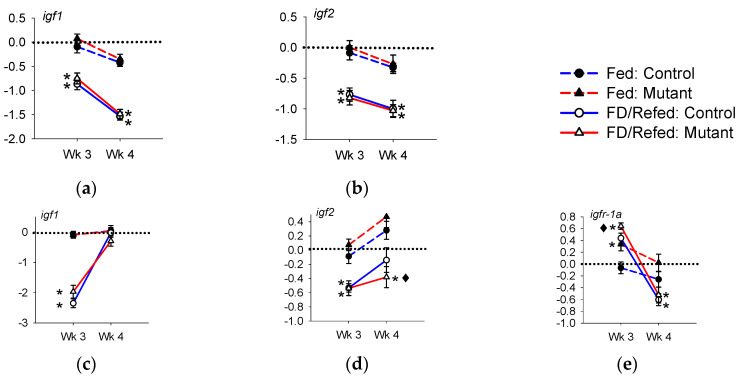
Expression of *igf1*, *igf2*, and *igfr-1a* in liver (**a**,**b**) and white muscle (**c**,**d**,**e**) during feed deprivation and refeeding. The y-axis represents gene expression values in arbitrary units normalized to the continuously fed control group from Wk 3 and expressed as log_2_ (fold change). The horizontal dotted line is a visual reference for log_2_ (fold change) = 0. Asterisks indicate that the feed-deprived (FD, Wk 3) or refed (Wk 4) groups (solid line) differ from continually fed fish (dashed line) within the same treatment group and week (*p* < 0.05, *n* = 21). The black diamond indicates that the fold change differs between the mutants and controls.

In liver, the expression of both *igf1* and *igf2* decreased during feed deprivation to approximately 0.5-fold that of continuously fed levels and remained at approximately this level after one week of refeeding; the pattern of regulation did not differ between the controls and IGFBP-2b mutants (Figure 5a,b, Table S2). In white muscle, the expression of *igf1* and *igf2* in both the controls and mutants was reduced to approximately 0.2-fold and 0.6-fold, respectively, that of the fed fish. During refeeding, the expression of *igf1* returned to baseline levels, as did *igf2* in controls, while the *igf2* expression in mutants remained low (Figure 5c,d, Table S3). In white muscle, the expression of *igfr-1a* was increased during feed deprivation (Figure 5e), which was up-regulated to a greater degree in the controls versus mutants (Table S3) and demonstrated an overshoot response during feed deprivation. 

#### 3.3.4. Expression of *igfbp* in Liver

**Figure 6:**
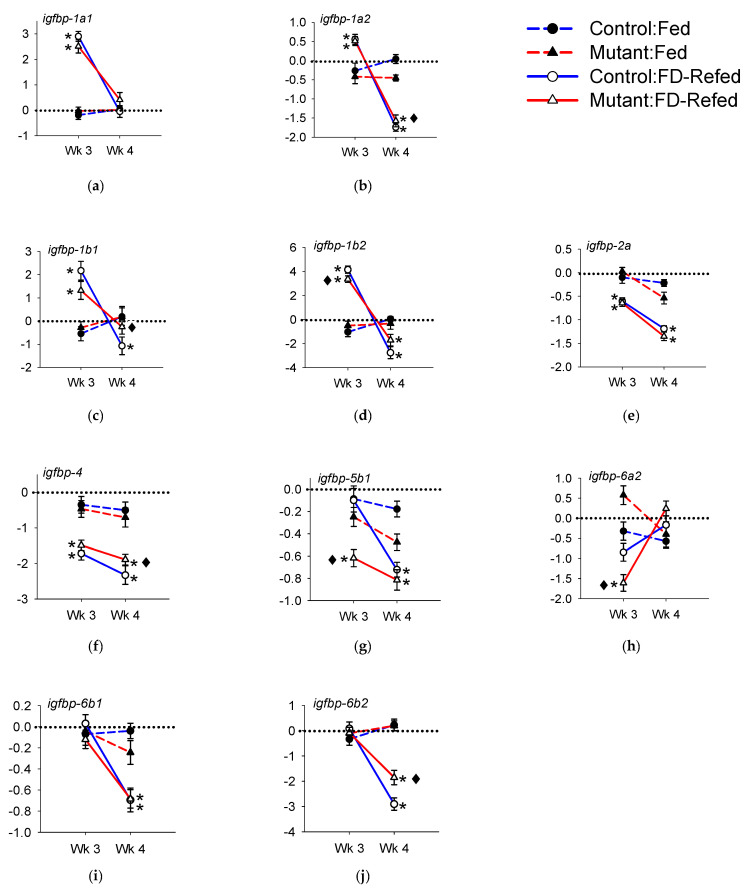
Hepatic expression of *igfbp* genes during feed deprivation (FD) and refeeding in controls (blue lines) and mutants (red lines). Genes are indicated in the top left corner of each panel (**a**–**j**). The y-axis represents gene expression values normalized to the continuously fed control group from week 3 and expressed as log_2_ (fold change). The horizontal dashed line is a visual reference for log_2_ (fold change) = 0. Asterisks indicate that the feed-deprived (FD, Wk 3) or refed (Wk 4) groups (solid line) differ from continually fed fish (dashed line) within the same treatment group and week (*p* < 0.05, *n* = 21). The black diamond indicates that the fold change differs between mutants and controls.

The expression of *igfbp* genes in liver is shown in Figure 6; comparison of the magnitude of regulation between control and mutant fish is provided in Table S2. During feed deprivation, the abundance of all *igfbp-1* transcripts increased by between 1.5–7.5-fold (Figure 6a–d), although up-regulation of *igfbp-1b2* (Figure 6d) was attenuated in mutants. During refeeding expression of *igfbp-1* genes either returned to that of continuously fed fish or demonstrated an overshoot response, although the magnitude of reduction was diminished for *igfbp-1a2* (Figure 6b) and *igfbp-1b1* (Figure 6c) in mutants. Expression of *igfbp-2a* was reduced to approximately 0.5-fold in both feed deprived and refed fish and was not affected by IGFBP-2b mutagenesis (Figure 6e). The approximately 50% reduction in *igfbp-4* during feed deprivation was sustained during refeeding, although down-regulation was more dramatic during refeeding in controls (Figure 6f). In mutants expression of *igfbp-5b1* was reduced 25% during feed deprivation and remained low throughout refeeding while controls exhibited reduced expression just during refeeding (Figure 6g). For *igfbp-6a2*, expression decreased during feed deprivation in mutants (to 0.17-fold) and returned to fed levels upon refeeding; in controls expression levels were not affected by feeding treatments (Figure 6h). For both *igfbp-6b1* and *igfbp-6b2*, expression decreased during refeeding only, but *igfbp-6b2* exhibited the greatest reduction in controls (90%) upon refeeding (Figure 6i,j).

#### 3.3.5. Expression of *igfbp* in White Muscle

**Figure 7:**
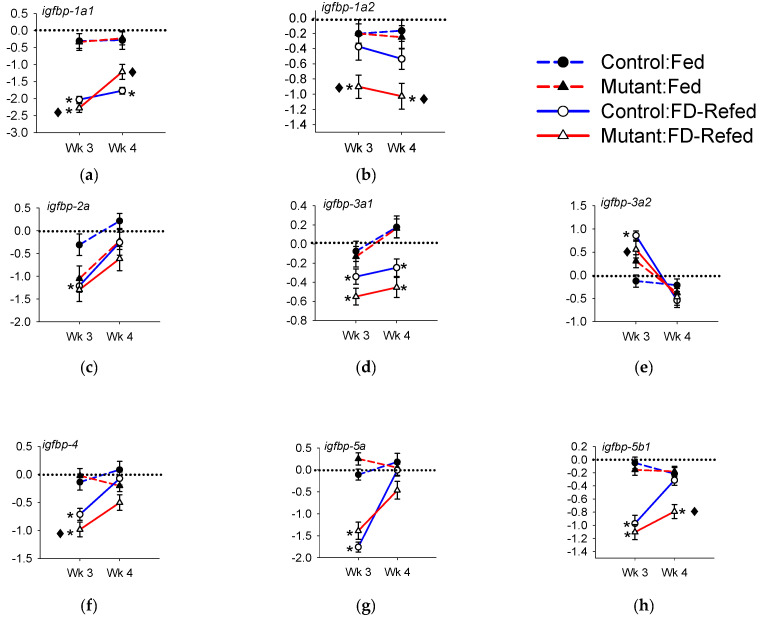

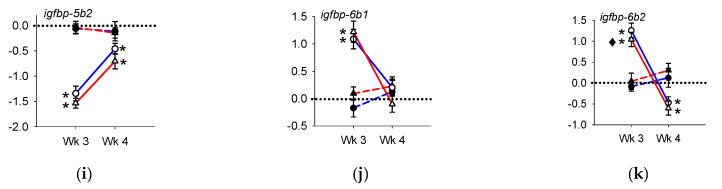
White muscle expression of *igfbp* genes during fed deprivation (FD) and refeeding in controls (blue lines) and mutants (red lines). Genes are indicated in the top left corner of each panel (**a**–**k**). The y-axis represents gene expression values normalized to the continuously fed control group from week 3 and expressed as log_2_ (fold change). The horizontal dashed line is a visual reference for log_2_ (fold change) = 0. Asterisks indicate that the feed deprived (FD, Wk 3) or refed (Wk 4) groups (solid line) differ from continually fed fish (dashed line) within the same treatment group and week (*p* < 0.05, *n* = 21). The black diamond indicates the fold change differs between mutants and controls.

The expression of *igfbp* genes in white muscle is shown in Figure 7; comparison of the magnitude of regulation between control and mutant fish is provided in Table S3. The expression of *igfbp-1a1* was reduced during feed deprivation, but the magnitude of the reduction was greater in mutants (0.19-fold) versus controls (0.24-fold) (Figure 7a). Reduced *igfbp-1a1* expression in controls held steady throughout refeeding, while the expression in mutants increased to a similar level compared to continuously fed fish. The expression of *igfbp-1a2* was reduced to approximately 0.50-fold throughout feed deprivation and refeeding in IGFBP-2b mutants; expression in controls was not significantly different from fed levels (Figure 7b). In feed deprived control fish, *igfbp-2a* was down-regulated to 0.43-fold of fed levels and increased upon refeeding while in mutants expression remained similar to fed fish (Figure 7c). During both feed deprivation and refeeding, expression of *igfbp-3a1* was reduced to approximately 0.70-fold of fed fish and was not different between controls and mutants (Figure 7d). In contrast, expression of *igfbp-3a2* increased 1.8-fold in controls during feed deprivation before changes in feed intake (Figure 7e). The abundance of *igfbp-4* transcript decreased during feed deprivation, but to a greater extent in mutants than in controls, before returning to levels similar to fed fish during refeeding (Figure 7f). The expression of all igfbp-5 genes decreased during feed deprivation to similar magnitudes in controls and mutants and approached fed levels during refeeding (Figure 7i). The only difference between controls and mutants occurred for *igfbp-5b1*, in which controls returned to fed levels at a faster rate than mutants (Figure 7h). Abundance of *igfbp-6b* transcripts responded similarly, with increases during feed deprivation and either a return to baseline (*igfbp-6b1*) or an overshoot response (*igfbp-6b2*) during refeeding (Figure 7j,k). Differential expression between controls and mutants occurred as an attenuated up-regulation of *igfbp-6b2* in mutants (Figure 7k).

### 3.4. Fifteen-Month Post Hatch

#### 3.4.1. Free Versus Total Serum IGF-I, and Serum IGFBPs

Older cohorts (~15 month post hatch) of fish from the previous study were used for the analysis of total versus free serum IGF-I. As in the younger fish, the body weight and fork length did not differ between controls and mutants, and mutants exhibited reduced concentrations of serum IGF-I (Table 1). However, the concentrations of free serum IGF-I were similar between groups (mean: 0.28 ng/mL), as was the ratio of free to total IGF-I (0.16%) (Table 1). Serum IGFBP-2b levels in mutants were significantly lower than those in controls. Serum 32-kDa IGFBP levels were similar between groups. 

#### 3.4.2. Correlations among IGF and IGFBP

Total and free IGF-I in serum showed no relationships with serum IGFBP-2b abundance in controls or mutants (Table 2). On the other hand, serum total and free IGF-I were negatively correlated with serum 32-kDa IGFBP abundance in mutants. In mutants, there was a positive relationship between total IGF-I and free IGF-I levels, although such a relationship was not seen in controls. In controls, there was a positive relationship between serum 32-kDa IGFBP and IGFBP-2b abundance.

## 4. Discussion

In this study, gene editing was successful at reducing the abundance of IGFBP-2b in serum by greater than 80% compared to pair-injected controls. This response is likely primarily a result of disruption of the IGFB-2b1 gene, since *igfbp-2b2* transcript was not present at detectable levels in either liver or white muscle. Higher expression of the *igfbp-2b1* gene relative to *igfbp-2b2* is also reported in Atlantic salmon [14]. Despite the considerable reduction in this IGFBP of greatest abundance in serum, IGFBP-2b mutants did not exhibit a morphological response with respect to body weight, fork length, or growth response. However, the components of the IGF/IGFBP system were uniquely regulated in IGFBP-2b mutants during continuous feeding, feed deprivation, and refeeding. These findings suggest that differential regulation of the IGF/IGFBP system in mutants maintains IGF-I signaling at a level comparable to controls to support a similar growth potential. This discussion will primarily focus on the unique regulation of the IGF/IGFBP system in mutants as a compensatory response to the loss of IGFBP-2b, rather than address the regulation of IGFBP by changes in nutrient supply, which has been previously characterized in salmonids [13,17,18,22,32,33,34,35].

During continuous feeding (i.e.: basal growth), IGFBP-2b mutants consistently exhibited 20–35% less serum IGF-I compared to the controls, despite exhibiting similar levels of hepatic *igf1* expression. These responses provide strong support for the notion that reduction in serum IGF-I is primarily a function of increased IGF-I degradation and/or secretion rather than reduced hepatic synthesis. In coho salmon, the molar ratio between serum IGF-I and IGFBP-2b is estimated at 1:1.5~2.8 [24]. If this ratio is applied to rainbow trout, the 83% reduction in IGFBP-2b in mutants would tilt this ratio in favor of IGF-I (1:0.26~0.48). In teleost fish, IGFBP-2b is functionally homologous to IGFBP-3 in mammals, both binding approximately 80% of total circulating IGF-I [9,36], although in mammals this occurs as a ternary complex between IGF-I, IGFBP-3, and the acid labile subunit (ALS) [10]. In humans, unbound IGF-I has an estimated half-life of less than 10 minutes; this increases to 25 minutes by binding to an IGFBP and to approximately 16 hours in the ternary complex with IGFBP-3 and the ALS [11,37]. Deletion of the IGFBP-3 gene in mice decreased IGF-I half-life and reduced serum IGF-I concentrations by 40–60% [38,39,40]. Therefore, the reduction in serum IGF-I appears to be a conserved response to loss of the most abundant serum IGFBP, supporting that these proteins are central regulators of total serum IGF-I levels in vertebrates, potentially by extending its half-life in circulation. In the current study, a positive correlation between serum IGFBP-2b and IGF-I abundance in controls from the feeding study further supports this mechanism, which appeared to be disrupted in IGFBP-2b mutants. The consequence may be a direct flux of IGF-I from the protein-bound to the free form; this concept is supported by the positive relationship between total and free IGF-I in mutants, as well as the reduction in serum IGF-I. It is of note that free IGF-I levels and free/total IGF-I ratios were similar between the controls and mutants, which emphasizes the importance of compensatory responses by other IGFBPs and IGF-IRs to maintain the IGF-I signaling.

An additional IGFBP band at 32 kDa detected in trout serum may play a role in carrying and delivering circulating IGF-I to target tissues by cooperating with IGFBP-2b. The fact that serum 32-kDa IGFBP abundance correlated with serum total IGF-I in the feeding experiment suggests that it participates in transporting total IGF-I. In addition, serum 32-kDa IGFBP abundance showed a positive relationship with serum IGFBP-2b, which suggests their regulatory and/or functional interaction. We hypothesize that IGFBP-2b is the major IGF-I carrier present in a molar excess to IGF-I, and 32-kDa IGFBP is less abundant but capable of carrying IGF-I and limiting the availability of circulating IGF-I to target tissues when released from IGFBP-2b. The hypothesis comes from findings that in mutants of 15 month post hatch, the reduction in the serum total IGF-I was not as large as that of IGFBP-2b and serum 32-kDa IGFBP abundance was negatively related to total and free IGF-I levels which were tightly linked in such a situation. Although we are aware that it is speculative, our findings warrant future study using the IGFBP-2b mutant trout to unravel the mechanisms of growth regulation by IGF-I and IGFBPs in fish. The identity of the 32-kDa IGFBP is not known at present and thus another subject of future study.

Comparable to the current findings, two studies in mice report that loss of IGFBP-3 does not affect growth rate [38,40], although improved growth with IGFBP-3 knockout is also reported [39] and the overexpression of IGFBP-2b in zebrafish reduced embryonic growth [41]. Compensatory responses in rainbow trout with reduced IGFBP-2b include decreased expression of hepatic *igfbp-1a2*, although this response was not detected by the ligand binding assay, and increased expression of muscle *igfr-1a*. In fish and mammals, IGFBP-1 is recognized as growth-inhibitory, since it competitively binds to the IGF receptor [42] to prevent ligand binding and increases during catabolic conditions [43]. In masu salmon, serum IGFBP-1a levels are negatively correlated with individual growth rate [21], supporting that the down-regulation in mutants is beneficial for growth potential. In muscle, the only IGFBP that was differentially regulated between continuously fed controls and mutants was *igfbp-2a*. This protein is generally regarded as growth-inhibitory in fish, since body weight decreases in zebrafish and mice over-expressing IGFBP-2a and IGFBP-2, respectively [41,44]. There is consensus in mammals that IGFBP-2 bound to extracellular matrix proteins serves as an active reservoir for locally produced IGFs [45], therefore down-regulation of *igfbp-2a* in mutants may represent a compensatory mechanism to increase the capacity for interaction between IGF receptors and muscle-derived IGF-I. Collectively, the down-regulation of these inhibitory IGFBP in mutants supports increased IGF-I signaling; this may offsets reductions in serum IGF-I and activate metabolic and mitogenic mechanisms required for growth during continuous feeding.

Additional genes that were differentially regulated between fed mutants and controls include hepatic *igfbp-5b1* and *igfbp-6a2*. The physiological significance of these responses is less clear, since the functional roles of these binding proteins in this tissue have not been fully characterized in fish. In mice, the abundance of IGFBP-5 in serum doubled with loss of IGFBP-3 [39], likely a compensatory response to stabilize serum IGF-I, since IGFBP-5 can also form a complex with IGF-I and the ALS. However, this IGFBP-5 function does not directly translate to fish, since the ALS protein is not detected in serum. In salmonids, IGFBP-5 is regarded as a growth-promoter in muscle, since its expression typically increases with anabolic treatments in myocyte cultures [33,46], although it is unknown how these responses translate to hepatocytes. However, in human-derived hepG2 cells IGFBP-5 can promote glucose uptake and regulate lipid metabolism [47], suggesting there could be differences in nutrient metabolism between controls and mutants, despite similar growth performance. The potential implications for increased regulation of *igfbp-6a2* in liver are also speculative, since the function of IGFBP-6, a protein with a higher affinity for IGF-II than IGF-I in mammals, is not well defined in fish [48]. However, unique to fish is the potentially significant role that IGF-II plays in post-embryonic growth mechanisms that parallel those of IGF-I [22,49]. Therefore, up-regulation of *igfbp-6a2* may function to inhibit IGF-II signaling in mutants, although the physiological effect of increased expression is unclear. 

The patterns of gene expression during feed deprivation and refeeding provide strong support for IGFBP and locally produced IGFs as significant regulators of IGF signaling [17,22]. During feed deprivation, the expression of growth-inhibitory IGFBP (hepatic *igfbp-1* and muscle *igfbp-6*) increased, while anabolic components (muscle *igf1*, *igf2*, *igfbp-5*, *igfbp-4*) decreased with either returns to baseline or an overshoot response during refeeding. While these responses were generally observed in both controls and mutants, the magnitude of up- or down-regulation often differed, although the weight loss responses were similar between treatment groups. These findings support the notion that both the controls and mutants reduced IGF-I signaling to similar levels during feed deprivation, although variable regulation of the IGF/IGFBP system components may have compensated for the reduction in serum IGF-I and IGFBP-2b in mutants. Specifically, the up-regulation of hepatic *igfbp-1b* transcripts was tempered in mutants, potentially due to the low concentrations of serum IGF-I and therefore a reduced need for additional sequestration by IGFBP-1b. Similarly, the down-regulation of *igfbp-1a* genes that inhibit local IGF-I binding to its receptor [50] was more dramatic in mutant muscle. The reduction in *igfbp-3a1* in muscle was also greater in mutants; the significance of this response is unclear, since the role of IGFBP-3 in fish is not well defined and its function in mammals as the major IGF-I carrier is not homologous in teleosts [13,32]. In addition, the up-regulation of *igfbp-6a2* expression was attenuated in mutants, although this may reflect the diminished inhibition of IGF-II signaling more than IGF-I signaling. Collectively, these findings suggest that the negative regulation of IGF-I signaling is attenuated during feed deprivation in mutants, potentially to offset the further reduction in serum IGF-I.

Contrary to the continuous feeding and feed deprivation treatment groups, the differential expression of IGF/IGFBP system components during refeeding did not support an enhanced IGF-I signaling capacity in mutants. In liver of the controls, there was an overshoot response for inhibitory IGFBP (*igfbp-1a2*, *igfbp-1b1*, and *igfbp-1b2*), where expression was down-regulated beyond levels in continually fed fish, leading to an increased capacity for IGF-I signaling to support recovery growth. In mutants, the overshoot response did not occur for *igfbp-1b1* and was attenuated for *igfbp-1a2* and *igfbp-1b2*, suggesting there may be less support for recovery growth. In addition, in control fish the abundance of serum IGFBP-2b increased to basal levels during refeeding, while levels remained very low in mutants, likely driven more by gene mutagenesis than regulation of expression. Similarly, the down-regulation of anabolic *igf2* and *igfbp-5b1* during feed deprivation in control muscle returned to basal levels after one week of refeeding, whereas the expression in mutants remained low. The anabolic effects of IGF-I and IGF-II in rainbow trout myocyte culture include stimulation of glucose uptake and DNA synthesis and activation of the PI3K/Akt signaling pathway [49], suggesting that the rapid up-regulation of gene expression during refeeding is central for recovery growth. Supporting the role of IGF-II during recovery growth is the regulation of the *igfbp-6* transcripts (IGF-II-inhibitory) that were up-regulated in muscle during feed deprivation and either returned to basal levels (*igfbp-6b1*) or demonstrated an overshoot response (*igfbp-6b2*) during refeeding. However, the response of the *igfbp-6* transcripts was similar between mutants and controls, suggesting that any variation in IGF-II signaling occurs via differential regulation of *igf2* expression. Regardless, differences in the regulation of the hepatic *igfbp-1* genes and *igf2* and *igfbp-5b1* in white muscle suggest a comparatively reduced capacity to stimulate IGF-I signaling during refeeding in mutants. Supporting this concept was a numerically reduced SGR (albeit not significant, *p* = 0.09) in mutants compared to controls. This finding is consistent with IGFBP-2b being important for recovery growth, a deficiency that could not be overcome in mutants by the compensatory regulation of other IGFBP.

## 5. Conclusions

In this study, we demonstrate the use of gene editing technology to disrupt the expression of the duplicated IGFBP-2b gene in rainbow trout. The findings support that in salmonids the primary function of IGFBP-2b is to bind serum IGF-I, ultimately affecting IGF-I half-life and circulating IGF-I concentrations. Despite reductions in serum IGF-I due to the loss of IGFBP-2b, growth performance or weight loss did not differ between controls and mutants, due in part to the compensatory regulation of 32 kDa IGFBP and IGFBP genes, as well as the IGF-II and IGF receptor. We conclude that there was a coordinated regulation of IGF/IGFBP system components in mutants that collectively maintained IGF-signaling at a level comparable to the controls that was commensurate with nutrient intake. 

## Figures and Tables

**Table 1:** Indices of growth performance and serum IGF-I in fed fish harvested at 15 months post hatch. Different superscript letters indicate that means differ between controls and mutants (*p* < 0.05, *n* = 15). ADU: arbitrary density units.

Variable	Controls	Mutants	PSEM	*p*-Value
body weight (g)	722.0	622.1	50.3	0.310
fork length (mm)	355.5	341.7	8.2	0.361
total serum IGF-I (ng/mL)	184.9 ^a^	147.3 ^b^	11.5	0.035
free serum IGF-I (ng/mL)	0.26	0.31	0.08	0.867
free/total IGF-I (%)	0.16	0.17	0.04	0.925
serum IGFBP-2b (ADU)	1483 ^a^	270 ^b^	136	<0.001
serum 32 kDa IGFBP (ADU)	964	1108	95	0.476

**Table 2:** Pearson correlations between the serum variables in controls and mutants at 15 months post-hatch. The top value in each cell is the correlation coefficient; the bottom is the *p*-value (*n* = 15). *P*-values < 0.05 are shown in bold.

	Controls			Mutants		
	Free IGF-I	IGFBP-2b	32 kDa IGFBP	Free IGF-I	IGFBP-2b	32 kDa IGFBP
total IGF-I	0.037 0.897	0.127 0.651	0.171 0.542	0.861 **<0.001**	0.432 0.132	−0.803 **<0.001**
free IGF-I		−0.078 0.780	−0.353 0.196		0.298 0.347	−0.752 **0.005**
IGFBP-2b			0.813 **<0.001**			−0.283 0.328

## Supplementary Materials

The following is available online at https://www.mdpi.com/2073-4425/11/12/1488/s1: Table S1: Primers used for PCR and RT-PCR; Table S2: Fold changes in serum variables and gene expression in liver; Table S3: Fold changes in gene expression in white muscle.
