# Supplementary material for: Compensatory Response of the Somatotropic Axis from IGFBP-2b Gene Editing in Rainbow Trout (Oncorhynchus mykiss)
Source: Genes (Basel). 2020 Dec 10;11(12):1488. doi: 10.3390/genes11121488 (PMC7763687; doi:10.3390/genes11121488)
Supplement: Supplementary file 1 [file genes-11-01488-s001.zip › Table S1.docx]

| Table S1. Primers (5’ – 3’) used for PCR and RT-PCR. Efficiencies are not indicated when transcript abundance could not be accurately quantified. | | | | | |
| --- | --- | --- | --- | --- | --- |
| Oligo | Sequence (5’-3’) | Accession Number | Product Size (bp) | Primer Efficiency | |
|  |  |  |  | Liver | Muscle |
| PCR Primers |  |  |  |  |  |
| IGFBP-2b1 | TTAGCTGCGGTTTGTTTCTG | NM_001124557.1 | 326 |  |  |
|  | GCGGTTGGGTACTGTGTCC |  |  |  |  |
| IGFBP-2b2 | TGCTAACACAGTTGAAGGTTC | JX674936.1 | 428 |  |  |
|  | TGGGTACCGGGTCTACTTTG |  |  |  |  |
| TYR2 | TTCTTCTGGGTGTGTTGGGT | NM_001124222.1 | 268 |  |  |
|  | ATGTGCGGTTGTAGAAGGC |  |  |  |  |
| RT-PCR Primers |  |  |  |  |  |
| *igf1* | GCTGCAGTTTGTGTGTGGAG | NM_001124696.1 | 124 | 1.88 | 1.87 |
|  | TAGCTCGCAACTCTGGAAGC | XM_021577176.1 |  |  |  |
| *igf2* | ATTGCGCTGGCACTTACTCT | NM_001124697.1 | 176 | 1.87 | 1.84 |
|  | CACTCCTCCACGATACCACG |  |  |  |  |
| *igfr-1a* | CCTGCCCAGCGACTTTGACT | XM_036954830.1 | 231 | - | 1.87 |
|  | ATGTTATTGCCACGGCGGAT |  |  |  |  |
| *igfbp-1a1* | CAAAACCCCGAACAAGGAGC | NM_001278935.1 | 73 | 1.97 | 1.93 |
|  | TGGAGCCGGAGTCTGATACC |  |  |  |  |
| *igfbp-1a2* | GAGAAGCTGAGCGAGTGTCC | XM_021599617.1 | 151 | 1.90 | 2.48 |
|  | GGGTGCACCTCAGTCCATAC |  |  |  |  |
| *igfbp-1b1* | AGAGCTGGGGATCCTAGACC | NM_001124561.1 | 182 | 2.05 | - |
|  | ACCTTGGCCTTGATGCTCTC |  |  |  |  |
| *igfbp-1b2* | GGATAGCCAGCTCTCAGCAG | XM_021589286.1 | 234 | 2.15 | - |
|  | TCGGAGCGTCCATGCTTTAG |  |  |  |  |
| *igfbp-2a* | AATTTAGCGGGGAGGTGGTG | *2a1*:NM_001124649.1 | 209 | 1.96 | 1.93 |
|  | CAGCTCCTGCTGACACTGAA | *2a2*:XM_021611002.1 |  |  |  |
| *igfbp-2b1* | CCACTGCCGTCCTTCAGATG | XM_021597998.1 | 159 | 2.04 | - |
|  | CAAGGGACAGGGAACGTAACA |  |  |  |  |
| *igfbp-2b2* | AGAAGGCGAATTCTGTGGCA | XM_021579985.1 | 224 | - | - |
|  | TGGTCGGCTTCTTTTGAGGG |  |  |  |  |
| *igfbp-3a1* | CAGTGTCTGGGGGAGTCAAC | XM_021604521.1 | 222 | - | 1.85 |
|  | GTGCACAAAGCAATACCACGA |  |  |  |  |
| *igfbp-3a2* | ATGCCCGGTCTATGCTTGTT | XM_021599618 | 136 | - | 1.92 |
|  | CTTTTGCCAGAGGCTTGCAC |  |  |  |  |
| *igfbp-3b1* | TTGCTGGTGTGTCGACAAGT | XM_021613488.1 | 217 | - | - |
|  | CCCAGCCATTTTGGACCCT |  |  |  |  |
| *igfbp-3b2* | AGCCATTGCCAGGTTTCGAC | XM_021589287.1 | 230 | - | - |
|  | CCATGCTCTGCCCTCCATAAG |  |  |  |  |
| *igfbp-4* | GAACGGAGACTTCCATGCCA | *4a1*:XM_021557125 | 142 | 1.90 | 2.02 |
|  | CATTAGCTGGTGGCAGTCCA | *4a2*:XM_021568020.1 |  |  |  |
| *igfbp-5a* | CAAGCTCCACTCTGTCAGCA | *5a1*:XM_021570672.1 | 247 | - | 1.97 |
|  | CACCAAAACGGTCCACACAC | *5a2*:XM_021611004.1 |  |  |  |
| *igfbp-5b1* | AGGTTTAACAATCGCGAGACG | NM_001124652 | 170 | 2.11 | 2.06 |
|  | GGCTCCTTCACGAGTTGACA |  |  |  |  |
| *igfbp-5b2* | CGTTTATTTTGACTGAAGTGTGCTG | XM_021598000.1 | 228 | - | 1.89 |
|  | CACGGCTCGCATGGTACATA |  |  |  |  |
| *igfbp-6a1* | ACACCCAACACCCAGTGAAG | XM_021608285.1 | 135 | - | - |
|  | CATGCTTGTCACAGTTGGGC |  |  |  |  |
| *igfbp-6a2* | GGTCACTGCTGGTGTGTGAA | XM_021569058.1 | 156 | 1.82 | - |
|  | TCCTCTCGATCCTCACCCAT |  |  |  |  |
| *igfbp-6b1* | TCTGTTGGTGTGTGGACGAG | NM_001124560.1 | 192 | 1.60 | 1.93 |
|  | CTGTCGAGGCAGTGTCTAGC |  |  |  |  |
| *igfbp-6b2* | ACGATTGAACCGGCTAAGCA | XM_021565958.1 | 246 | 2.08 | 1.97 |
|  | AGCCAGAATCCAAGATCCGC |  |  |  |  |
