# Supplementary material for: Compensatory Response of the Somatotropic Axis from IGFBP-2b Gene Editing in Rainbow Trout (Oncorhynchus mykiss)
Source: Genes (Basel). 2020 Dec 10;11(12):1488. doi: 10.3390/genes11121488 (PMC7763687; doi:10.3390/genes11121488)
Supplement: Supplementary file 1 [file genes-11-01488-s001.zip › Table S2.docx]

|  | **Feed Deprivation** | | | **Refeeding** | | |
| --- | --- | --- | --- | --- | --- | --- |
| **Gene/Protein** | **Controls** | **Mutants** | **PSEM** | **Controls** | **Mutants** | **PSEM** |
| IGF-I | 0.36 | 0.26 | 0.12 | 0.41 | 0.39 | 0.02 |
| IGFBP-2b | 0.65 | 0.43 | 0.08 | 0.78 ^b^ | 2.75 ^a^ | 0.25 |
| 32 kDa IGFBP | 0.03 | -- | 0.02 | 0.48 | 0.67 | 0.11 |
| *igf1* | 0.55 | 0.54 | 0.05 | 0.45 | 0.43 | 0.03 |
| *igf2* | 0.59 | 0.53 | 0.05 | 0.61 | 0.56 | 0.04 |
| *igfbp-1a1* | 7.46 | 5.21 | 1.31 | 0.86 | 1.04 | 0.33 |
| *igfbp-1a2* | 1.47 | 1.64 | 0.16 | 0.27 ^b^ | 0.47 ^a^ | 0.04 |
| *igfbp-1b1* | 4.53 | 2.27 | 1.97 | 0.23 ^b^ | 0.53 ^a^ | 0.17 |
| *igfbp-1b2* | 17.75 ^a^ | 8.22 ^b^ | 2.96 | 0.12 | 0.17 | 0.10 |
| *igfbp-2a* | 0.66 | 0.61 | 0.03 | 0.50 | 0.56 | 0.03 |
| *igfbp-4* | 0.30 | 0.40 | 0.04 | 0.23 ^b^ | 0.36 ^a^ | 0.05 |
| *igfbp-5b1* | 0.93 ^a^ | 0.75 ^b^ | 0.05 | 0.67 | 0.73 | 0.04 |
| *igfbp-6a2* | 0.56 ^a^ | 0.17 ^b^ | 0.09 | 1.17 | 1.14 | 0.21 |
| *igfbp-6b1* | 1.02 | 0.91 | 0.06 | 0.62 | 0.68 | 0.06 |
| *igfbp-6b2* | 1.06 | 0.85 | 0.26 | 0.09 ^a^ | 0.19 ^b^ | 0.04 |

**Table S2.** Fold change in serum IGF-I or IGFBP abundance and hepatic gene expression compared to continuously fed fish of the same treatment group. Different letters indicate the magnitude of regulation differs between controls and mutants within the same feeding treatment.
