# Supplementary material for: Compensatory Response of the Somatotropic Axis from IGFBP-2b Gene Editing in Rainbow Trout (Oncorhynchus mykiss)
Source: Genes (Basel). 2020 Dec 10;11(12):1488. doi: 10.3390/genes11121488 (PMC7763687; doi:10.3390/genes11121488)
Supplement: Supplementary file 1 [file genes-11-01488-s001.zip › Table S3.docx]

|  | **Feed Deprivation** | | | **Refeeding** | | |
| --- | --- | --- | --- | --- | --- | --- |
| **Gene** | **Controls** | **Mutants** | **PSEM** | **Controls** | **Mutants** | **PSEM** |
| *igf1* | 0.20 | 0.26 | 0.04 | 0.84 | 0.74 | 0.18 |
| *igf2* | 0.69 | 0.63 | 0.04 | 0.69 ^a^ | 0.49 ^b^ | 0.13 |
| *igfr-1a* | 1.36 ^a^ | 1.17 ^b^ | 0.07 | 0.72 | 0.62 | 0.05 |
| *igfbp-1a1* | 0.24 ^a^ | 0.19 ^b^ | 0.02 | 0.25 ^b^ | 0.42 ^a^ | 0.10 |
| *igfbp-1a2* | 0.77 ^a^ | 0.57 ^b^ | 0.07 | 0.71 ^a^ | 0.51 ^b^ | 0.07 |
| *igfbp-2a* | 0.43 | 0.82 | 0.12 | 0.62 | 0.64 | 0.30 |
| *igfbp-3a1* | 0.79 | 0.71 | 0.05 | 0.70 | 0.62 | 0.05 |
| *igfbp-3a2* | 1.81 ^a^ | 1.09 ^b^ | 0.15 | 0.74 | 0.81 | 0.11 |
| *igfbp-4* | 0.61 ^a^ | 0.47 ^b^ | 0.05 | 0.80 | 0.77 | 0.08 |
| *igfbp-5a* | 0.30 | 0.29 | 0.04 | 0.73 | 0.61 | 0.09 |
| *igfbp-5b1* | 0.51 | 0.50 | 0.05 | 0.89 ^a^ | 0.64 ^b^ | 0.05 |
| *igfbp-5b2* | 0.39 | 0.33 | 0.04 | 0.69 | 0.64 | 0.06 |
| *igfbp-6b1* | 2.13 | 2.04 | 0.36 | 0.87 | 0.70 | 0.08 |
| *igfbp-6b2* | 2.39 ^a^ | 1.69 ^b^ | 0.31 | 0.54 | 0.47 | 0.07 |

**Table S3.** Fold change in gene expression in white muscle compared to continuously fed fish within the same treatment group. Different letters indicate the magnitude of regulation differs between controls and mutants within the same feeding treatment.
